# Antibacterial and Antifungal Management in Relation to the Clinical Characteristics of Elderly Patients with Infective Endocarditis: A Retrospective Analysis

**DOI:** 10.3390/antibiotics11070956

**Published:** 2022-07-15

**Authors:** Camelia Melania Budea, Marius Pricop, Felix Bratosin, Iulia Bogdan, Miriam Saenger, Ovidiu Ciorica, Laurentiu Braescu, Eugenia Maria Domuta, Mirela Loredana Grigoras, Cosmin Citu, Mircea Mihai Diaconu, Iosif Marincu

**Affiliations:** 1 Department of Ear-Nose-Throat, “Victor Babes” University of Medicine and Pharmacy Timisoara, Eftimie Murgu Square 2, 300041 Timisoara, Romania; cameliafizedean@yahoo.com; 2 Methodological and Infectious Diseases Research Center, Department of Infectious Diseases, “Victor Babes” University of Medicine and Pharmacy, Eftimie Murgu Square 2, 300041 Timisoara, Romania; felix.bratosin7@gmail.com (F.B.); iulia.georgianabogdan@gmail.com (I.B.); miriam.saenger@googlemail.com (M.S.); laurbraescu@gmail.com (L.B.); edomuta@uoradea.ro (E.M.D.); grigoras.mirela@umft.ro (M.L.G.); imarincu@umft.ro (I.M.); 3 Discipline of Oral and Maxillo-Facial Surgery, Faculty of Dental Medicine, “Victor Babes” University of Medicine and Pharmacy Timisoara, Eftimie Murgu Square 2, 300041 Timisoara, Romania; 4 Business Administration and Economics Faculty, West University of Timisoara, Johann Heinrich Pestalozzi Street 16, 300115 Timisoara, Romania; ovidiu.ciorica@e-uvt.ro; 5 Department of Cardiovascular Surgery, Institute for Cardiovascular Diseases, Str. Gh. Adam nr. 13A, 300310 Timisoara, Romania; 6 Surgery Department, Faculty of Medicine and Pharmacy, University of Oradea, Piata 1 Decembrie 10, 410073 Oradea, Romania; 7 Department of Anatomy and Embryology, “Victor Babes” University of Medicine and Pharmacy, Eftimie Murgu Square 2, 300041 Timisoara, Romania; 8 Department of Obstetrics and Gynecology, “Victor Babes” University of Medicine and Pharmacy Timisoara, Eftimie Murgu Square 2, 300041 Timisoara, Romania; citu.ioan@umft.ro (C.C.); diaconu.mircea@umft.ro (M.M.D.)

**Keywords:** endocarditis, bacterial infections, fungal infections, antibiotics, antifungals

## Abstract

Infective endocarditis (IE) is increasingly prevalent in the elderly, particularly due to the rising frequency of invasive procedures and intracardiac devices placed on these individuals. Several investigations have highlighted the unique clinical and echocardiographic characteristics, the microorganisms implicated, and the prognosis of IE in the elderly. In addition, the old population seems to be fairly diverse, ranging from healthy individuals with no medical history to patients with many ailments and those who are immobile. Furthermore, the therapy of IE in this group has not been well investigated, and worldwide recommendations do not propose tailoring the treatment approach to the patient’s functional state and comorbid conditions. A multicenter research study was designed as a retrospective study of hospitalized patients with infective endocarditis, aiming to examine the characteristics of elderly patients over 65 years old with infective endocarditis in relation to the antibiotic and antifungal treatments administered, as well as to quantify the incidence of treatment resistance, adverse effects, and mortality in comparison to patients younger than 65. Based on a convenience sampling method, we included in the analysis a total of 78 patients younger than 65 and 131 patients older than 65 years. A total of 140 patients had endocarditis on native valves and 69 patients had endocarditis on prosthetic valves. A significantly higher proportion of elderly patients had signs of heart failure on admission, and the mortality rate was significantly higher in the elderly population. A majority of infections had a vascular cause, followed by dental, maxillo-facial, and ENT interventions. The most common complications of IE were systemic sepsis (48.1% of patients older than 65 years vs. 30.8% in the younger group). The most frequent bacterium involved was *Staphylococcus aureus*, followed by *Streptococcus* spp. in a total of more than 50% of all patients. The most commonly used antibiotics were cephalosporins in 33.5% of cases, followed by penicillin in 31.2% and glycopeptides in 28.7%, while Fluconazole was the initial option of treatment for fungal endocarditis in 24.9% of cases. Heart failure at admission (OR = 4.07), the development of septic shock (OR = 6.19), treatment nephrotoxicity (OR = 3.14), severe treatment complications (OR = 4.65), and antibiotic resistance (OR = 3.24) were significant independent risk factors for mortality in the elderly patients. Even though therapeutic management was initiated sooner in the older patients, the associated complications and mortality rate remained significantly greater than those in the patients under 65 years old.

## 1. Introduction

The elderly have a known risk factor for infection owing to a naturally impaired immunity caused by aging and the effects of other medical conditions. Respiratory infectious diseases are within the primary ten factors of mortality among the elderly. Until 2050, the world population aged 80 years and older is anticipated to expand by more than three times up to approximately 400 million [1,2]. More than half of older persons in high-income nations have three or more chronic diseases, and, as fatality drops, those with multiple comorbid conditions are expected to live longer; therefore, as life expectancy rises, infections in the elderly are becoming more significant [3,4]. Not only does the prognosis of infections in the elderly rely on the organism involved, but it often relies often on the patients’ conditions, the degree of treatment reliance, the nutritional state, and the cognitive capacity. A geriatric examination may identify common health issues in elderly adults, such as cognitive impairment, delirium, falls, and urine incontinence, with onset or exacerbation when infections occur [5].

Infective endocarditis (IE) is a life-threatening illness with significant mortality and complication rates in the affected patients—mostly in older patients with coexisting conditions. It is characterized by the infection of the endocardial layer, often involving the valves due to a high turbulent flow [6], and affects around 5–15 per 100,000 individuals annually [7]. Despite major advances in diagnosis and medical care, the death rates have remained high, hovering around 40 percent one year following the acute infection [8]. There are exceedingly varied and non-specific manifestations on top of the notable cardiac presentation which can be associated with the immunological and embolic consequences of IE, affecting the brain, skin, bones, joints, kidneys, and eyes, among the most frequently involved organs [9,10,11,12,13]. This systemic diversity might result in a large delay in the diagnosis of IE, severely impacting the prognosis of patients [14,15]. Despite significant advances in diagnosis and therapy, the fatality rates continue to be high [16,17,18].

Research indicates that the proportionate rise in the prevalence of IE was the greatest among the senior population aged 65 years or older [19]. It has been shown that the risk of endocarditis for older people is almost five times higher than that of the general population. The high frequency of undetected degenerative valve disease and the rising use of invasive procedures and implanted medical devices may be examples of such reasons [20], which may also affect the prognosis of older people with IE [21]. Several procedures require antibiotic prophylaxis for patients with cardiac conditions who are at a high risk for infective endocarditis, including maxillo-facial procedures and non-dental invasive procedures of the upper and lower gastrointestinal tract, genitourinary and gynecological procedures, and those of the upper and lower respiratory tract, including ear, nose, and throat (ENT) procedures and bronchoscopy [22,23].

Recent studies have attempted to define the features of IE in old patients, but contradictory findings have been reported, such as the belief that IE in the elderly has distinctive clinical features compared to IE in younger patients [24,25]. The etiology of IE seems to be influenced by the higher prevalence of certain risk factors among older persons, such as prosthetic valves or implantable devices [26,27]. Transesophageal echocardiography has been discovered to greatly boost the diagnostic sensitivity for IE in older individuals, allowing for immediate antimicrobial management and assuming a higher death rate due to comorbidities and the restricted utilization of surgical therapy in this population [28,29]. In contrast to these findings, it has been postulated that epidemiologic variables may have a bigger role in determining the clinical presentation, the echocardiographic characteristics, the incidence of complications, and the necessity of surgery [30,31]. Therefore, the current study aimed to observe the characteristics of patients over 65 years old who are affected by infective endocarditis in association with the antibiotic and antifungal treatment used. A secondary target was to determine the frequency of treatment resistance, treatment complications, and mortality in these patients, as compared to those younger than 65.

## 2. Results

### 2.1. General Characteristics of the Study Participants

A total of 78 cases of infective endocarditis were found in adults younger than 65 years, and 131 were found in the elderly, with an average age of 59.6 years in the first group and 67.5 years in the second (*p*-value < 0.001). The majority of patients were men—55.1% among those younger than 65 years and 51.8% among the group of older patients. It was observed that 55.1% of the full cohort of patients were overweight (BMI > 25 kg/m^2^). The tobacco and alcohol use inquiry determined that approximately 30% of all patients were smokers, and 4% used to consume alcohol on a daily basis. The comorbidities involved the cardiovascular system in more than 40% of the cohort, followed by digestive and metabolic diseases, without a significant difference between the groups, as seen in Table 1.

### 2.2. Endocarditis Features

An in-depth analysis of the endocarditis patient outcomes is presented in Table 2. The median time taken from symptom onset until treatment was about three days in the younger patients and two days in the elderly. About 80% of all patients were observed to have vegetations on the ultrasound examination, and close to 20% had the presence of a cardiac abscess. A total of 140 patients had endocarditis on native valves, and 69 had endocarditis on prosthetic valves, with a statistically significant difference between the study groups (*p*-value = 0.040). The most common valve involved was mitral—it was involved in about 45% of all patients. The majority of infections had a vascular cause, followed by dental, maxillo-facial, and ENT interventions. The third-most-common cause of infection was a gastrointestinal source.

The etiology, procedures, and interventions of the patients with infective endocarditis presented in Table 3 showed that a total of 57.3% of patients older than 65 required surgical intervention of the involved valve, compared to 42.3% of the younger patients (*p*-value = 0.036), while the younger patients were significantly more likely to be referred for valve repair instead of valve replacement (36.4% vs. 24.0%, *p*-value = 0.044). The most common complications of IE were systemic sepsis (48.1% of patients older than 65 years vs. 30.8% in the younger group, *p*-value = 0.014). It was observed that a significantly higher proportion of elderly patients had signs of heart failure on admission (51.9% vs. 32.1%, *p*-value = 0.005). The severity of valvular regurgitation was also significantly higher in the elderly group, where 45.0% had a moderate level of regurgitation compared with 28.2% in the younger patients (*p*-value = 0.010). Oxygen supplementation was required in a significantly higher proportion of elderly patients (65.6%), while 60.3% were admitted to the ICU during hospitalization. The duration of the ICU stay was significantly longer in the patients older than 65 years (7.7 days vs. 5.9 days of hospitalization in the ICU, *p*-value < 0.001). Lastly, the mortality rate was significantly higher in the elderly population (40.5% vs. 26.9%, *p*-value = 0.047) (Table 3).

### 2.3. Microbal Identification and Antibacterial and Antifungal Management

**Figure 1:**
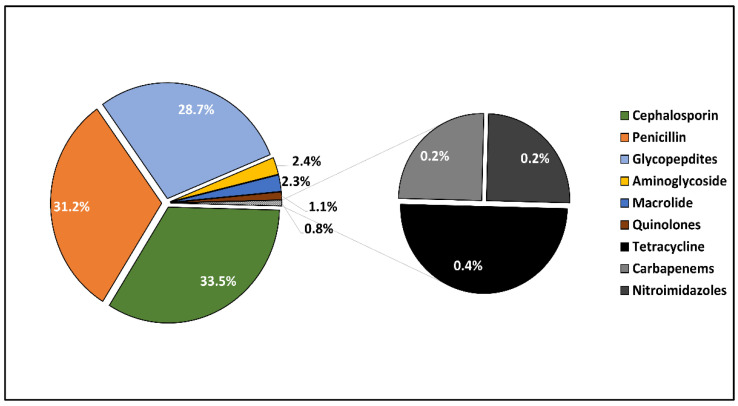
Distribution of antibiotics used for patients with endocarditis. Data are represented as a pie chart of the nine antibiotic classes used in the affected patients in descending order of frequency of use.

**Figure 2:**
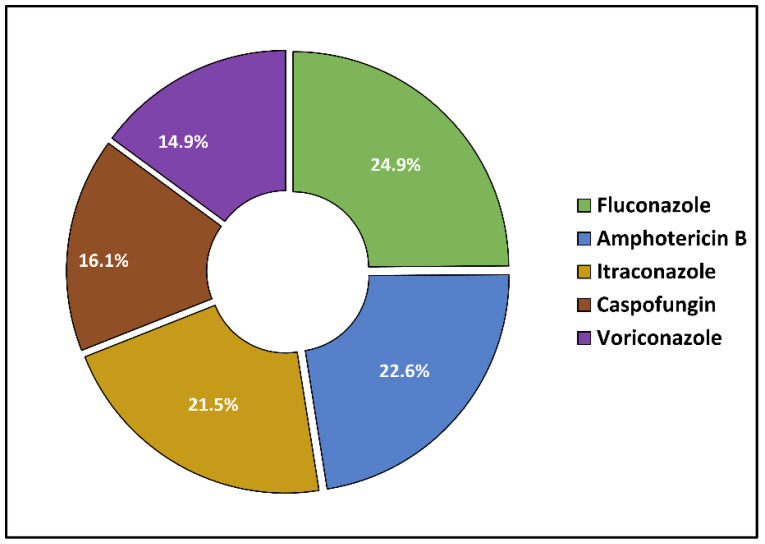
Distribution of antifungals used among patients with endocarditis. Data are represented as a pie chart of the five antifungals used in the affected patients in descending order of frequency of use.

Table 4 describes the microbial identification and the antibacterial and antifungal management. The etiologic diagnosis of endocarditis was performed by a conventional culture in about 60% of cases, followed by 25% involving PCR tests, while the remaining 15% of patients were tested by both culture and PCR. It was observed that 85% of the cases had a bacterial origin, and the remaining 15% were cases of fungal endocarditis. The most frequent bacterium involved was *Staphylococcus aureus*, followed by *Streptococcus* spp. in a total of more than 50% of all patients. Other bacteria involved were CoNs and *Enterococcus faecalis*. A total of 19 patients had candida endocarditis, and 9 were identified as having *Aspergillus*. There was a significant difference in severe treatment complications; 17.6% of the elderly were affected compared with 7.7% of the younger patients (*p*-value = 0.046). The most commonly used antibiotics were cephalosporins (in 33.5% of cases), followed by penicillin (in 31.2%) and glycopeptides (in 28.7%), as seen in Figure 1. The antifungals used are presented in Figure 2; Fluconazole was the initial option of treatment for fungal endocarditis in 24.9% of cases (Figure 2).

It was observed that the patients older than 65 were significantly more likely to develop medication antibiotic side effects such as nephrotoxicity; 13.7% of the elderly were affected, compared with 5.1% among those younger than 65. Regarding the nephrotoxic effect in the elderly, it was observed that six patients taking cephalosporins had a kidney injury as a side effect, followed by five patients under glycopeptide therapy. The most common cause of nephrotoxicity among the elderly under antifungal treatment was amphotericin B in four patients, followed by four cases of kidney injury in the patients taking azoles and caspofungin-induced renal failure in three patients. Other significant side effects among the elderly were enterocolitis, liver injury, delirium, and falls, the first being the most common side effect in both study groups, albeit in a significantly higher proportion in the elderly group (29.0% vs. 14.1%, *p*-value = 0.013), as presented in Table 5. The most common causes of enterocolitis in the elderly were penicillin and cephalosporins. In the same manner, antifungals caused a significantly higher proportion of falls and delirium among the elderly (20.6% vs. 0.0%, *p*-value = 0.037).

### 2.4. Risk Factor Analysis

The risk factor analysis presented in Table 6 identified, via multiple logistic regression analysis, that heart failure at admission, the development of septic shock, treatment nephrotoxicity, severe treatment complications, and antibiotic resistance were significant independent risk factors for mortality in both the young and old patients, although the likelihood (OR) was higher in the patients older than 65.

## 3. Discussion

This multicentric study managed to provide a comprehensive analysis of infective endocarditis cases from western Romania during a four-year period and described in detail the antibiotic and antifungal management of the affected patients, along with the treatment complications and risk factors for mortality. Set in the global context of infective endocarditis, the data from Romania are limited to clinical monocentric studies [32,33], while country-wide epidemiologic data and real-world statistics are scarcely reported or lacking. Therefore, a real estimate of the recent evolution of cases over time is a difficult estimate in the country. However, during the four-year period of the data analysis, nearly two-thirds (62.6%) of the participants in our research were older than 65. In older patients, the incidence of endocarditis was described to be about nine times greater compared to that in younger patients, although the threshold for old age is a matter of constant change and debate [32,33,34]. The age distribution of endocarditis is also changing, as endocarditis mostly impacted young individuals with rheumatic valvular disease, but it now primarily affects elderly patients, as rheumatic heart disease is widely prevented through prophylaxis methods [35]. The patients are older because the survival rate of patients with rheumatic and congenital heart disease has increased, and degenerative valvular disease is common among the elderly [36].

In the majority of patients, the main source of infection had a vascular origin. Similar findings were reported by other studies that discovered that vascular sources of infection were present in 63% of healthcare-associated infections [37]. The second most common cause of infection was comprised of maxillofacial, dental, and ENT interventions, which is likely due to improper prophylaxis in high-risk patients or incorrect sterile equipment usage, as documented by previous investigators [38]. However, the identification of the infection source was difficult in many cases since one-third of the patients had negative blood cultures. Similar research reported blood culture positive rates ranging from 40 to 70 percent [39,40].

Similar to the current findings, *Staphylococcus* spp. was also the most commonly detected microbe in the European Infective Endocarditis Registry (EURO ENDO), involved in more than 40% of the cases observed, followed by *Streptococcus* and *Enterococcus* spp., which were found with a smaller frequency than that in our research [41]. It is believed that the frequency of *Staphylococcus aureus* infectious endocarditis has grown, and in the industrialized world, it has become the most prevalent causal organism [42]. For example, in a comprehensive study conducted in India, it was identified that *Staphylococcus* spp. was the predominant cause, whereas rheumatic heart disease was the most prevalent cardiac substrate at risk [43]. In our investigation, *Staphylococcus aureus* was identified more often in individuals with prosthetic valve endocarditis due to the propensity of this bacterium to attach to prosthetic materials. According to several previous works of research, *Staphylococcus aureus* is the primary cause of prosthetic valve endocarditis, and it was observed that *Streptococcus* strains, mainly *Streptococcus bovis*, are the most prevalent strains discovered in prosthetic valve endocarditis, while *Staphylococcus* spp. were second in terms of frequency [44,45].

Echocardiography is the most often utilized imaging technique for diagnosis; therefore, transthoracic ultrasound was conducted on all patients, while transesophageal ultrasound was performed in selected patients with valvular prosthesis. Other findings suggest that more patients should receive transesophageal echocardiography even in the absence of prosthetic valve involvement [46]. In the majority of patients with extensive vegetations and intracardiac abscess, vegetations served as the most important diagnostic criteria, as studies report that at least 60–70 percent of patients show vegetations and that close to 20% of patients have cardiac abscess, similarly to our findings [47,48].

In our analysis, the most common consequences were heart failure and acute renal failure, as almost one-third of patients were diagnosed with congestive heart failure, whilst approximately 13% of patients were diagnosed with cardiogenic shock and/or severe pulmonary edema at admission, in conjuncture with the epidemiologic studies reporting a decrease in the prevalence of congestive heart failure and cardiogenic shock. Acute renal failure was found in 26.9 percent of the patients who participated in this trial, much greater than the 17.7 percent of patients in the EURO ENDO study. In the EURO ENDO registry, up to 40 percent of patients had embolic events as the most common complication, whereas 44.1 percent were diagnosed with a stroke following infective endocarditis. Our analysis found a lower incidence of embolic events. The IE etiology of gram-negative bacilli related to septic shock in our patients, and this finding was also noted in other studies in which more than 40% of patients with this etiology were in septic shock [49]. In spite of breakthroughs in diagnosis and treatment, the disease’s incidence and mortality did not decline, and the disease’s death rate had reached 20% in the previous 30 years [50].

In our investigation, a significant mortality rate was seen in 26.9% of the patients under 65 years old and 40.5% of those older than 65. Some writers also report significant death rates between 15 and 30% [51]. It was generally observed that mortality is closely correlated with the etiology of endocarditis, it having the highest rates in fungal endocarditis [52]. The optimal antifungal treatment is still a matter of debate. The *Candida* species may build biofilms on native and artificial heart valves, which can impair the antifungal effectiveness of antifungal medications used to treat *Candida endocarditis*. Voriconazole is effective against a broad range of clinically significant fungal infections, such as *Candida*, *Aspergillus*, and *Fusarium*. Amphotericin B has been used to treat endocarditis caused by *Aspergillus*. Amphotericin B is less toxic than traditional amphotericin and may be provided at greater dosages, making it particularly useful for patients with compromised renal function or who develop nephrotoxicity when taking standard amphotericin [53]. Amphotericin B cannot successfully penetrate and treat FE-associated vegetations on its own. Itraconazole and Caspofungin are effective against resistant *Aspergillus* infections. Compared to other antifungal medicines, echinocandins are less toxic and have fewer drug interactions. They are equally effective as Amphotericin B in treating non-neutropenic individuals infected with *Aspergillus* [54].

Even though timely antibacterial and antifungal management can be initiated, there are multiple consequences associated with medical treatment in the elderly. As renal function diminishes with age, elderly people also have a reduced renal clearance that may raise the risk of nephrotoxicity or systemic side effects when renally cleared antimicrobials are chosen without dosage modification, as observed in our patients. Clinicians should be aware that nephrotoxic antibiotics such as aminoglycosides and vancomycin are more likely to induce acute kidney failure in older patients with diabetes or who take diuretics and ACE inhibitors [55]. Another antibiotic, Daptomycin, a quickly bactericidal cyclic lipopeptide, has been approved for the treatment of right-sided staphylococcal endocarditis and seems to be well tolerated by the elderly, although it is much more costly than vancomycin because of its proven efficacy against bacterial biofilm. It might be regarded as the treatment of choice in patients with significantly compromised renal function and in cases of IE affecting cardiac implanted electronic devices [56].

Considering the scarcity of data and epidemiological studies about infective endocarditis in Romania, this research brings important new information about the evolution of such cases and their treatment in a multicentric setting. Therefore, the current study’s main strength is the detailed analysis of IE during the last four years in western Romania. Nevertheless, several limitations are worth mentioning. First of all, the retrospective design relying strongly on patient recordkeeping and the quality of data digitally copied from paper records can allow for human errors while reporting the data. Another limitation is the sample size that was restricted by the time spread of the retrospective analysis and the participation of the four clinics involved in the treatment of IE from the western Romanian region; therefore, the current results might not confidently represent the characteristics and outcomes of all patients affected in the country of interest.

## 4. Materials and Methods

### 4.1. Study Design and Ethical Considerations

A multicenter research study was designed as a retrospective study of hospitalized patients with infective endocarditis. The setting comprised three tertiary hospitals in Western Romania, where patients were admitted to the Cardiology and Internal Medicine Departments in the period starting from January 2018 until December 2021. The research protocol was approved by the Ethics Committee of the “Victor Babes” University of Medicine and Pharmacy of Timisoara, Romania and by the Ethics Committee of the hospitals included in the current study.

### 4.2. Inclusion Criteria and Study Variables

We used a convenience sampling approach to determine the ideal sample size, which was determined to be at least 385 patients, for a 5% margin of error at a 95% level of confidence and an approximate incidence of 3–10 in 100,000 patients per year [57]. A database and patient paper record search were conducted to determine the cases of infective endocarditis from patients admitted to the three hospitals participating in the current study. The diagnosis of infective endocarditis was made by the use of electrocardiograms, imagistic findings (cardiac transthoracic or transesophageal ultrasound and computed tomography), and bacterial identification by conventional cultures and PCR. Other detailed assessments were performed to determine the clinical evolution of affected patients and to observe whether organ failure occurs. Patients older than 65 who were confirmed as having endocarditis were included in the elderly group “≥65 years”, while those who were younger than 65 were defined as adults “<65 years”. Patients with incomplete records or those lacking consent were removed from the study. Other patient-specific characteristics were retrieved from paper and database records, such as the recent history of dental, maxillo-facial, and ENT procedures and the history of valvuloplasty or valve replacement surgery.

The variables considered to be relevant to the current study comprised the following: (1) patient background analysis: age, sex, body mass index, tobacco and alcohol use, and comorbidities; (2) treatment options and patient outcomes: days from symptom onset until hospitalization, localization on native and prosthetic valves, tests performed for infection identification, the presence of heart failure on admission, the severity of valvular regurgitation, oxygen supplementation, and outcomes (intensive care unit admission, days spent in the ICU, days between symptom onset and death, days between symptom onset and ICU admission, mortality, and days until discharge); (3) etiology, procedures, interventions, and complications; (4) microbial identification, antibacterial and antifungal management, and medical treatment complications; (5) risk factor analysis.

### 4.3. Statistical Analysis

The statistical analysis was conducted using IBM SPSS version 27 (IBM Corp, Armonk, NY, USA) and MedCalc for Windows, version 20 (MedCalc Software, Ostend, Belgium). This determined the absolute and relative frequencies of the categorical variables. The Chi-square test and Fisher’s test were used to compare proportions, and the Mann–Whitney test was used to compare nonparametric group differences. Using the Student’s t-test, the mean and standard deviations of continuously distributed values with a normal distribution were compared (unpaired, independent samples). In conclusion, a multivariate analysis adjusted for confounding variables was conducted to identify the independent risk factors for death. The significance criterion was established at a 0.05 alpha value.

## 5. Conclusions

Endocarditis affects the elderly disproportionately compared to younger patients, although endocarditis of artificial valves and endocarditis associated with medical therapy and vascular interventions are on the rise regardless of patient age. Other significant sources of infection were maxillo-facial surgery and ENT procedures, which were more likely to be associated with *Streptococcus* spp. and extensive vegetations on ultrasonography and were associated with an increased risk of cardioembolic events. Blood cultures that are negative are often seen despite this. *Staphylococcus aureus* is associated with large vegetations and intracardiac abscesses and is more frequent in prosthetic valve inflammatory endocarditis. A *Staphylococcus aureus* infection is severe, with outcomes such as congestive heart failure and embolic events. Additionally, *Staphylococcus aureus* seems to be related to mortality. *Streptococcus* spp. were often exacerbated by dense vegetation and stroke, while enterococcus infections were more prevalent in patients with multiple comorbidities. It was discovered that a greater incidence of gram-negative bacillus was related with a severe clinical course in the elderly, which was often exacerbated by septic shock compared to the younger patients. Antifungal management showed a greater toxicity than the antibiotics used in the studied patients, although the infection severity and mortality were higher in patients with fungal infections.

## Figures and Tables

**Table 1:** Background characteristics of the study participants.

Variables *	<65 Years (*n* = 78)	>65 Years (*n* = 131)	*p*-Value
**Age (mean ± SD)**	59.6 ± 7.2	67.5 ± 11.8	<0.001
**Sex**			0.651
Men	43 (55.1%)	68 (51.9%)	
Women	35 (44.9%)	63 (48.1%)	
**BMI**			
Underweight (<18.5 kg/m^2^)	5 (6.4%)	7 (5.3%)	0.051
Normal weight (18.5–25.0 kg/m^2^)	40 (51.3%)	46 (35.1%)	
Overweight (>25.0 kg/m^2^)	33 (42.3%)	59.5 (59.5%)	
**Tobacco and alcohol use**			
Smoking	28 (35.9%)	40 (30.5%)	0.423
Chronic alcohol consumption	3 (3.8%)	6 (4.6%)	0.800
**Comorbidities**			
Cardiac	33 (42.3%)	61 (46.6%)	0.549
Metabolic	13 (16.7%)	29 (22.1%)	0.339
Cerebrovascular	21 (26.9%)	48 (36.6%)	0.148
Digestive & liver	14 (17.9%)	34 (26.0%)	0.183
Kidney disease	6 (7.7%)	14 (10.7%)	0.476
Cancer	7 (9.0%)	18 (13.7%)	0.304

* Data are reported as *n* (%) and were calculated using the Chi-square test and Fisher’s exact test unless specified differently. BMI—Body Mass Index.

**Table 2:** Endocarditis features of the patients included in the study.

Variables *	<65 Years (*n* = 78)	>65 Years (*n* = 131)	*p*-Value
Days from symptom onset until treatment (median, IQR)	3 (2)	2 (2)	0.622
Presence of vegetations	61 (78.2%)	109 (83.2%)	0.369
Presence of cardiac abscess	14 (17.9%)	28 (21.4%)	0.550
**Localization on native valves (*n* = 140)**	59 (75.6%)	81 (61.8%)	0.040
Aortic	11 (18.6%)	33 (40.7%)	0.005
Aortic and tricuspid	16 (27.1%)	12 (14.8%)	0.072
Mitral	20 (33.9%)	24 (29.6%)	0.591
Mitral and aortic	12 (20.3%)	12 (14.8%)	0.391
**Localization on prosthetic valves (*n* = 69)**	19 (24.4%)	50 (38.2%)	0.040
Aortic biologic valve	3 (15.8%)	6 (12.0%)	0.676
Aortic mechanical valve	3 (15.8%)	7 (14.0%)	0.850
Mitral biologic valve	4 (21.1%)	12 (24.0%)	0.795
Mitral mechanical valve	5 (26.3%)	15 (30.0%)	0.763
Mitral and aortic biologic valve	2 (10.5%)	6 (12.0%)	0.864
Mitral and aortic mechanical valve	2 (10.5%)	4 (8.0%)	0.739
**Etiology**			
Peripheral/central vein catheter	27 (34.6%)	51 (38.9%)	0.532
Hemodialysis	6 (7.7%)	11 8.4%)	0.856
Cardiac surgery	3 (3.8%)	7 (5.3%)	0.623
Angiography	5 (6.4%)	9 (6.9%)	0.897
Vascular surgery	8 (10.3%)	4 (3.1%)	0.030
Gastrointestinal	7 (9.0%)	12 (9.2%)	0.963
Maxillo-facial interventions	9 (11.5%)	16 (12.2%)	0.884
Ear-nose-throat interventions	13 (16.7%)	21 (16.0%)	0.904

* Data are reported as *n* (%) and were calculated using the Chi-square test and Fisher’s exact test unless specified differently; IQR—Interquartile Range.

**Table 3:** Procedures, complications, and outcomes.

Variables *	<65 Years (*n* = 78)	>65 Years (*n* = 131)	*p*-Value
**Surgical repair (*n* = 140)**	33 (42.3%)	75 (57.3%)	0.036
**Type of surgery performed**			0.044
Aortic valve replacement	6 (18.2%)	20 (26.7%)	
Mitral valve replacement	11 (33.3%)	13 (17.3%)	
Double valve replacement	4 (12.1%)	24 (32.0%)	
Mitral valve repair	12 (36.4%)	18 (24.0%)	
**Complications**			
**Heart failure on admission**			0.005
Yes	25 (32.1%)	68 (51.9%)	
No	53 (67.9%)	63 (48.1%)	
**Severity of valvular regurgitation**			0.010
Mild	49 (62.8%)	54 (41.2%)	
Moderate	22 (28.2%)	59 (45.0%)	
Severe	7 (9.0%)	18 (13.7%)	
**Oxygen supplementation**			
Yes	38 (48.7%)	86 (65.6%)	0.015
No	40 (51.3%)	45 (34.4%)	
Cardiogenic shock	7 (9.0%)	19 (14.5%)	0.241
Valvular leak	7 (9.0%)	25 (19.1%)	0.049
Stroke	8 (10.3%)	19 (14.5%)	0.375
Atrioventricular block	18 (23.1%)	49 (37.4%)	0.031
Kidney failure	14 (17.9%)	47 (35.9%)	0.005
Mediastinitis	8 (10.3%)	27 (20.6%)	0.052
Systemic sepsis	24 (30.8%)	63 (48.1%)	0.014
**Outcomes**			
ICU admission	30 (38.5%)	79 (60.3%)	0.002
Days in the ICU (mean ± SD)	5.9 ± 2.2	7.7 ± 4.0	<0.001 ^t^
Days between symptom onset and death (mean ± SD)	8.2 ± 6.6	4.7 ± 6.0	<0.001 ^t^
Days between symptom onset and ICU admission (mean ± SD)	5.8 ± 4.1	3.0 ± 5.3	<0.001 ^t^
Mortality	21 (26.9%)	53 (40.5%)	0.047
Days until discharge (mean ± SD)	13.8 ± 4.3	19.4 ± 7.1	<0.001 ^t^

* Data are reported as *n* (%) and were calculated using the Chi-square test and Fisher’s exact unless specified differently. ^t^—Unpaired Student’s t-test; SD—Standard Deviation; ICU—Intensive Care Unit.

**Table 4:** Microbial identification and antibacterial and antifungal management.

Variables *	<65 Years (*n* = 78)	>65 Years (*n* = 131)	*p*-Value
**Tests performed for infection identification**			0.579
Culture	46 (59.0%)	77 (58.8%)	
PCR	47 (28.2%)	31 (23.7%)	
Culture and PCR	72 (12.8%)	23 (17.6%)	
**Testing**			
Positive samples	48 (61.5%)	77 (58.8%)	
False negative result	30 (38.5%)	54 (41.2%)	
**Identification**			0.542
Bacterial	69 (88.5%)	112 (85.5%)	
Fungal	9 (11.5%)	19 (14.5%)	
**Pathogens involved**			0.319
*Staphylococcus aureus*	22 (28.2%)	38 (29.0%)	
CoNs	7 (9.0%)	19 (14.5%)	
*Streptococcus* spp.	20 (25.6%)	20 (15.3%)	
*Escherichia coli*	3 (3.8%)	9 (6.9%)	
*Enterococcus faecalis*	12 (15.4%)	17 (13.0%)	
Other gram-negative bacilli	5 (6.4%)	9 (6.9%)	
*Candida* spp.	6 (7.7%)	13 (9.9%)	
*Aspergillus* spp.	3 (3.8%)	6 (4.6%)	
**Severe treatment complications**	6 (7.7%)	23 (17.6%)	0.046
**Treatment regimen type**			0.417
Monotherapy	45 (57.7%)	68 (51.9%)	
Combined	33 (42.3%)	63 (48.1%)	
**Multidrug resistance**			0.758
Yes	24 (30.8%)	43 (32.8%)	
No	54 (69.2%)	88 (67.2%)	
**Number of pathogens identified**			0.566
Monoinfection	73 (93.6%)	125 (95.4%)	
Two pathogens	5 (6.4%)	6 (4.6%)	

* Data are reported as *n* (%) and were calculated using the Chi-square test and Fisher’s exact unless specified differently. CoNs—Coagulase-negative staphylococci.

**Table 5:** Antibacterial and antifungal treatment side effects.

Variables *	<65 Years (*n* = 78)	>65 Years (*n* = 131)	*p*-Value
**Antibiotics**			
Acute immune reactions	5 (6.4%)	6 (4.6%)	0.566
Delayed reactions	4 (5.1%)	4 (3.1%)	0.449
Nephrotoxicity	4 (5.1%)	18 (13.7%)	0.049
Neurotoxicity	2 (2.6%)	9 (6.9%)	0.177
Liver injury	3 (3.8%)	16 (12.2%)	0.041
Enterocolitis	11 (14.1%)	38 (29.0%)	0.013
Falls and delirium	1 (1.3%)	16 (12.2%)	0.005
**Antifungals**			
Acute immune reactions	1 (1.3%)	3 (2.3%)	0.606
Delayed reactions	0 (0.0%)	2 (1.5%)	0.272
Nephrotoxicity	2 (2.6%)	11 (8.4%)	0.091
Neurotoxicity	1 (1.3%)	4 (3.1%)	0.417
Liver injury	2 (2.6%)	9 (6.9%)	0.532
Enterocolitis	14 (5.1%)	10 (7.6%)	0.483
Falls and delirium	0 (0.0%)	7 (20.6%)	0.037

* Data are reported as *n* (%) and were calculated using the Chi-square test and Fisher’s exact unless specified differently.

**Table 6:** Identification of significant risk factors for mortality in the patients with endocarditis.

Factors *	<65 Years or (95% CI)	*p*-Value	>65 Years or (95% CI)	*p*-Value
Heart failure at admission	3.15 (2.87–5.21)	<0.001	4.07 (3.44–6.90)	<0.001
Septic shock	3.08 (2.66–6.09)	<0.001	6.19 (4.15–8.28)	<0.001
Treatment nephrotoxicity	1.66 (1.07–2.34)	0.001	3.14 (2.36–4.03)	0.001
Severe treatment complications	3.39 (2.25–5.11)	<0.001	4.65 (3.82–6.21)	<0.001
Antibiotic resistance	2.61 (1.71–4.06)	<0.001	3.24 (2.09–5.52)	<0.001

*—Adjusted by age, COVID-19 severity, and pulmonary diseases.

## Data Availability

The data are available on request.
